# The Growth Factors in Advanced Platelet-Rich Fibrin (A-PRF) Reduce Postoperative Complications after Mandibular Third Molar Odontectomy

**DOI:** 10.3390/ijerph182413343

**Published:** 2021-12-18

**Authors:** Anna Starzyńska, Magdalena Kaczoruk-Wieremczuk, Michele Antonio Lopez, Pier Carmine Passarelli, Paulina Adamska

**Affiliations:** 1 Department of Oral Surgery, Medical University of Gdańsk, 7 Dębinki Street, 80-211 Gdańsk, Poland; mkaczoruk@gumed.edu.pl (M.K.-W.); paulina.adamska@gumed.edu.pl (P.A.); 2 Unit of Otolaryngology, University Campus Bio-Medico, 00128 Rome, Italy; micheleantonio.lopez@gmail.com; 3 Department of Head and Neck and Sensory Organs, Division of Oral Surgery and Implantology, Institute of Clinical Dentistry, Catholic University of the “Sacred Heart”, Fondazione Policlinico Gemelli IRCCS, 00168 Rome, Italy; piercarmine.passarelli@unicatt.it

**Keywords:** growth factors, advanced platelet-rich fibrin, A-PRF, third molar extraction, impacted teeth, wound healing

## Abstract

Surgical removal of impacted mandibular third molars constitutes one of the most frequently performed procedures within oral surgery. This surgery procedure is associated with many post-operative complications. Advanced platelet-rich fibrin (A-PRF) belongs to the second generation of platelet concentrates and is rich in numerous growth factors. The aim of this study was to assess the influence of A-PRF on selected clinical features following the surgical removal of impacted mandibular third molars. The research was conducted on 100 generally healthy patients, who underwent a lower third molar odontectomy in Department of Oral Surgery, Medical University of Gdańsk, Poland, between 2018 and 2019. The research group consisted of 50 patients (immediate A-PRF socket filling) and control group (50 patients without A-PRF socket filling). During the study, the following clinical features were assessed: pain (visual analog scale), analgesics intake, the presence of trismus, edema, hematomas within the surrounding tissues (e.g., cheek), prevalence of pyrexia, dry socket, secondary bleeding, presence of hematomas, skin warmth in the post-operative area, and bleeding time observed by the patient were analyzed on the 3rd, 7th, and 14th day after the procedure. There was a significant association between A-PRF socket filling and pain intensity, the analgesics intake, trismus, and edema on the 3rd and the 7th day (*p* < 0.05). The presence of hematomas and skin warmth on the 3rd day after the surgery (*p* < 0.05) were also statistically associated with A-PRF use. The study showed that in reducing the incidence of postoperative complications, A-PRF was more important than the position of the tooth or the duration of the procedure. The growth factors in A-PRF reduce postoperative complications, such as pain, trismus, edema, analgesics intake, presence of hematomas, and skin warmth, after mandibular wisdom teeth odontectomy.

## 1. Introduction

Pain, edema, and trismus are main and the most commonly observed post-operative complications that may occur in patients who have undergone oral surgery procedures. The other clinical features that might be also noticed are as follows: suggillations or hematomas within the face or neck, prolonged bleeding from the alveolus, pyrexia, elevated skin temperature in the vicinity of the operating field, and in some cases also dry socket symptoms. These symptoms can be observed particularly frequently as a consequence of surgical removal of mandibular third molars—a procedure considered to be the one of the most difficult and time-consuming among all oral surgery procedures. At the same time, it constitutes one of the most frequently performed procedures within this specialty [1,2,3,4]. The factors that determine the difficulty of the surgical procedure of avulsion of the third molar are depth and space available for removal of the impacted mandibular third molar, the angulation of the tooth, root spacing, size of the bone septum, presence or absence of a dilated tooth follicle, periodontal space, bone density, and the relation to the inferior alveolar nerve. Other factors that influence the occurrence of non-infectious complications after this procedure are the experience of the surgeon, the choice of the operating method, the time of the procedure, and soft tissues trauma [5,6,7,8,9].

A-PRF (advanced platelet-rich fibrin) belongs to the second generation of platelet concentrates. This autologous material, consisting solely of patient’s own blood elements, without anticoagulants and thrombin, is obtained from an animal source or any other agents [10,11]. As a consequence of the above, it does not cause allergies and there is no risk of cross infection. It consists of a three-dimensional fibrin matrix, rich in platelets and leukocytes, containing cytokines, stem cells, and growth factors, constituting a biodegradable scaffold, which contributes to microvascularisation development and encourages epithelial cells migration to its surface [12,13]. Platelets, treated as a A-PRF key factor, stimulate angiogenesis by means of a release of growth factors, such as PD-EGF (platelet-derived epidermal growth factor), PDGF (platelet-derived growth factor), VEGF (vascular-endothelial growth factor), FGF (fibroblast growth factor), or TGF (transforming growth factor). Substantial amounts of PDGF-AB, TGFβ-1 (tumor necrosis factor β-1), and TSP-1 (thrombospondin-1) can be found in A-PRF. The presence of leukocytes guarantees higher content of pro- and anti-inflammatory mediators, TNFα (tumor necrosis factor α), and interleukins (IL-1β, IL-6, and IL-4). Leucocytes also release VEGF and TGFβ-1 [14,15,16,17]. In Choukroun’s protocol, there is also a greater amount of monocytes, which are supposed to release bone morphogenetic proteins BMP-2 and BMP-7 (bone morphogenic factor 2 and 7), as well as VEGF. Owing to lower centrifugation speed in comparison with PRP-obtaining (platelet-rich plasma) protocol, the PRF net formation is a result of a natural process of a coagulation cascade [11,18,19,20,21]. Lower centrifugation speed helps to build fibrin net endurance, which is connected with more efficient uptake of cells and cytokines during the centrifugation and has an influence on subsequent gradual release of the contained growth factors. The ability to release growth factors from 1 to 4 weeks guarantees longer period of healing stimulation than in case of platelet-rich plasma use, which releases all the growth factors within the moment of application. Proper tissue healing is the priority factor influencing therapeutic success. A-PRF contributes to healing acceleration, faster angiogenesis, and reduces the risk of inflammation within the area of its application. It leads to a decrease in intensity of the post-operative complications [18,19,20,21,22,23,24,25,26,27,28]. In dentistry, it is used to treat and stimulate the healing process after the free gingival graft, dry socket, or medication-related osteonecrosis of the jaw has been obtained [28,29,30]. The method, based on PRF socket filling, can be beneficial in decreasing pain related to the post-odontectomy healing process [31,32,33]. A lower risk of a wound infection is also observed. Hemostatic properties have also been proven [11,12,13].

The aim of this study was to investigate the presence and intensity of the selected symptoms following mandibular third molars odontectomy with A-PRF application for immediate socket filling when compared with the control group, who underwent the same procedure using a standard method, without the use of A-PRF. We tried to evaluate the effectiveness of A-PRF application on the alveolus and its influence on the pain intensity, the amount of edema, bleeding, the level of trismus, prevalence of dry socket, the skin temperature, and pyrexia.

## 2. Materials and Methods

The prospective study included 100 patients (18–47 years of age) who underwent a lower third molar odontectomy at the Department of Oral Surgery, Medical University of Gdańsk, Poland, between June 2018 and January 2019. Only patients who were generally healthy, not taking any medications, without a history of allergies or addictions (without smoking, alcohol, or drug addictions), and presenting good oral hygiene were included in the study. The official approval from the institutional ethics committee was obtained prior to the research (Independent Bioethics Commission for Research, Medical University of Gdańsk, number of approval NKBBN/211/2018). Verbal and written consents for the procedure and for the participation in the study were obtained. During the research, the anonymity of the patients was preserved.

All odontectomy procedures were performed by the same experienced surgeon (M.K.-W.). Before the procedure, all of the patients underwent a radiological examination to assess the position of the wisdom tooth. The teeth were evaluated in terms of the grade of tooth retention, the angulation using the Winter classification, the relationship to mandibular ramus and the second molar—the Pell and Gregory classification—as well as the Pedersen index [9]. Only horizontally and vertically retained teeth which required the mucoperiosteal flap with osteotomy and where the difficulty of surgical tooth extraction was assessed as moderately or very difficult according to Pedersen’s classification were included in the study. Uniform groups were established in terms of the size of the population. The research group consisted of 50 patients (immediate A-PRF socket filling) and control group of 50 patients (without A-PRF socket filling). The selection of patients for each group was random via coin toss and was carried out by a surgeon who was not involved in the study.

### 2.1. PRF Preparation

**Figure 1:**
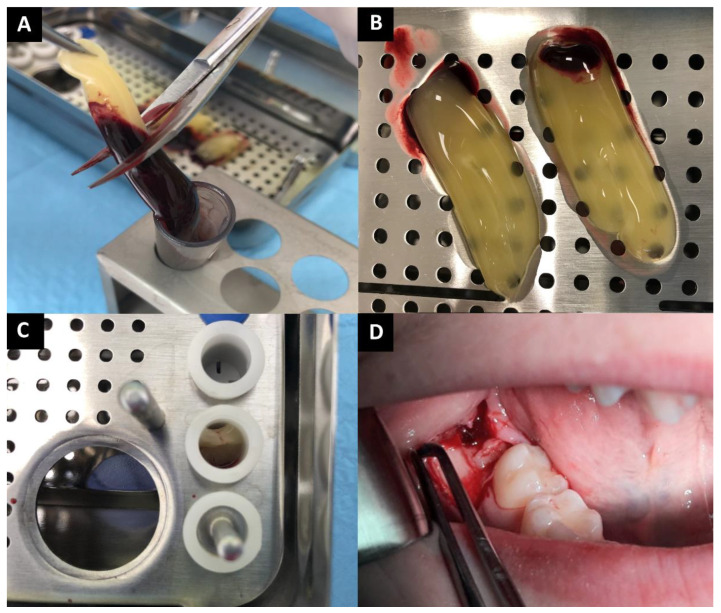
(**A**) Removing A-PRF clot from the tube; (**B**) A-PRF clots after remove from the tubes; (**C**) A-PRF corks preparation; (**D**) A-PRF application.

In the research group, before the surgical procedure began, 40 mL of venous blood was collected from the patients into 4 sterile, dry, anticoagulant-free, glass-coated plastic tubes (10 mL each) and immediately centrifuged (by the centrifuge All Centrifuge, Scilogex, LLC, Rocky Hill, CT, USA). The blood was collected up to 10 min before the procedure began (patient’s anesthesia). Due to the fact that anticoagulant is not present in the blood collection tube, the blood was centrifuged before it will start to coagulate. The time from blood collection to centrifugation was not longer than 2 min. Centrifugation time was 14 min and the speed was 1500 rpm. During the described process, 4 fibrin clots were obtained. A-PRF was dissected by scissors from red corpuscle base at the bottom, 2 mm below connection between layers, because this area is rich in platelets [3]. A-PRF clots were removed from the blood tubes and were put in special PRF Box. The material was formed into 4 corks and placed in the socket (Figure 1A–D). Prepared A-PRFs are usable up to 4 h from preparation, but in the case of this study, A-PRF clots were placed in the socket within 1.5 h from centrifugation.

### 2.2. Surgical Procedure

Before the surgery, local anesthesia was given via injection. Mandibular and buccal blocks were administered using 1.7 mL 4% articaine hydrochloride containing 1:100,000 epinephrine (Citocartin 100, Molteni Dental s.r.l., Scandicci (Florence), Italy). Triangle incision was made with no. 15 scalpel blade and the mucoperiosteal flap was raised. The osteotomy was performed using a round bur mounted on a W&H implanted surgical low-speed handpiece (Bürmoos, Austria), at 1200 rpm under abundant irrigation. Impacted third molar was removed by elevators and forceps. In the research group A-PRF was applied into the socket and in the control group the socket was left empty. In both groups, the flap was repositioned, and the wound was closed by 4–0 non-resorbable nylon sutures. Post-extraction instructions were given. All the patients were prescribed antibiotics (amoxicillin 0.875 g with clavulanic acid 0.125 g, every 12 h for 7 days) and analgesics (ketoprofenum 0.1 g).

### 2.3. Postoperative Evaluations

During the study, the following clinical features were assessed: pain (visual analog scale), analgesics intake (number of analgesics intake per day), the presence of trismus, edema, hematomas within the surrounding tissues (e.g., cheek), prevalence of pyrexia, dry socket, secondary bleeding, presence of hematomas, skin warmth in the post-operative area, and bleeding time observed by the patient. These were analyzed on the 3rd (48 h after the surgery), 7th, and 14th day after the procedure.

**Figure 2:**
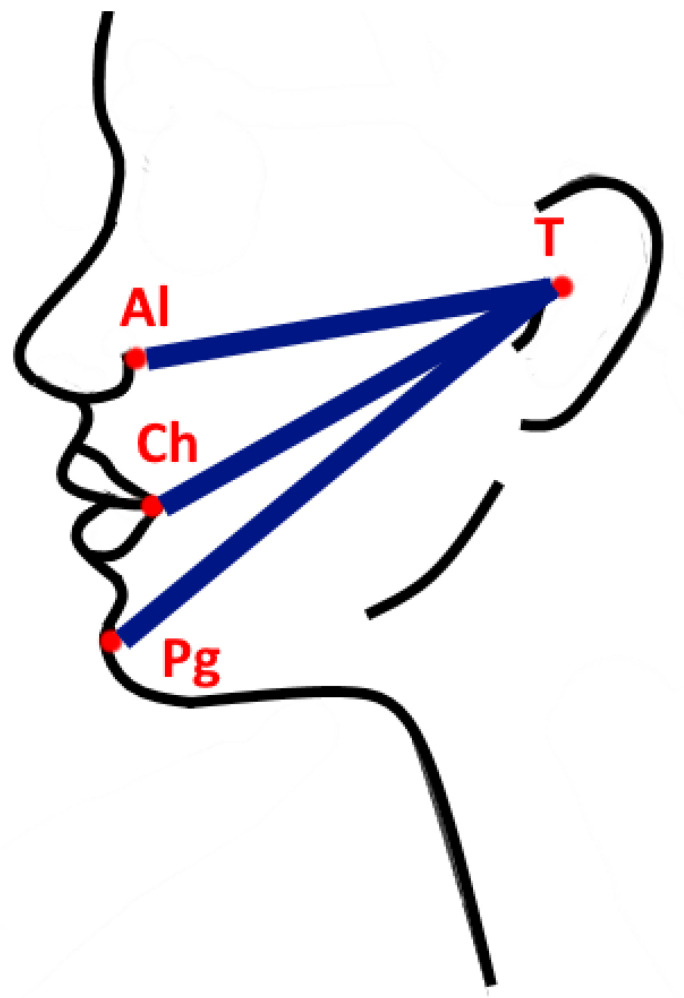
The location of the reference points within the skin and a schematic representation of the distance measurements between them.

Pain was assessed using an 11-point visual analog scale (VAS). A score of ‘0’ meant ‘no pain’; a score ‘10’ meant ‘very severe pain’. The patients were divided into 3 groups according to the intensity of the pain: mild (0–3), moderate (4–6), and severe (7–10). Patients were also asked about analgesics intake (number of the ingested tablets a day). Trismus was assessed by measuring the distance between upper and lower incisions, by the ruler. The first degree of trismus resulted in a mandibular abduction of 2.1–3.0 cm. The second degree of trismus was recorded when the patient abducted the mandible by 1.1–2.0 cm. The third degree of trismus was found when the jaw opening was limited to 0.1–1.0 cm. The fourth degree was the absence of trismus, i.e., the lower jaw abduction was more than 3.5 cm. Edema was evaluated by measuring the distances by elastic tape from the tragus (T) to 3 other anthropometric points (author’s own method): to the alare point (Al), to the chelion point (Ch), and to the soft tissue pogonion (Pg) (Figure 2). The distances were measured and recorded before the surgery, then measured during the follow-up visits and the measurements were compared. The studies distinguished three types of edema after the chiseling procedure, depending on its extent: (1) small edema—diagnosed during the follow-up examination when at least one of the measured distances between the reference points (T-Al, T-Ch, T-Pg) was greater by 5 to 15 mm than the baseline value (T0); (2) moderate swelling—at least one measurement from the three tested distances exceeded 15 mm in relation to the baseline value, but not more than 25 mm in comparison with the (T0) value; (3) large—at least one measurement at the control visit exceeded the difference of 25 mm with respect to the dimension T0. The cohesion of swelling was also assessed. Patients were asked about the bleeding time, which they observed between the end of surgery and the time when the bleeding stopped. They were also asked about secondary bleeding incident. The presence of hematoma within the surrounding skin layers was examined. The skin warmth in the post-operative area was assessed by symmetric palpation. The presence of dry socket was assessed on the basis of clinical examination and subjective symptoms reported by the patient. The temperature was measured at the follow-up visits.

### 2.4. Statistical Analysis

The statistical analysis was performed using the R statistical environment, R version 3.5.1. Ggplot2 package, STATISTICA 13.3 (TIBCO, Palo Alto, CA, USA) software and Excel 2016 (Microsoft Corporation, Fargo, NT, USA) was used for data visualization. The basic group characteristics were presented using descriptive statistics, with the use of two-way contingency tables for categorical variables and with the use of median and interquartile range for quantitative variables. The Shapiro–Wilk test was used for the comparison of the quantitative variables (measured on a ratio scale) between the groups. The variables measured on an ordinal scale and the variables measured on a ratio scale (non-normally distributed) were compared between the two groups using the Mann–Whitney—Wilcoxon test. Quantitative data variation through time was visualized using line charts. The relationship between two categorical variables was analyzed using either the Fisher or the chi-square test. The correlations between the variables, measured, at a minimum, on an ordinal scale, were analyzed using Kendall tau. A model of multivariate analysis of variance was performed, which examined the influence of A-PRF and selected variables (tooth position, degree of tooth retention, duration of the procedure) on postoperative complications. The significance level was determined as *p* < 0.05.

## 3. Results

### 3.1. Characteristic of Patients

The vast majority of the control and study group were women. The mean age between the groups was similar. The patients came mainly for the extraction of partially impacted teeth (82% in the control group vs. 94% in the study group). The vertical position of the third molar was predominant in the study group. Detailed clinical data are presented in Table 1.

Partially impacted teeth were more often in the vertical position (*p* = 0.005). Complete teeth retention was also associated with longer treatment duration (*p* = 0.048). Longer surgery time was noted in the case of horizontally positioned teeth (*p* = 0.069). Patients with complete tooth retention were younger compared with patients with partial retention (*p* = 0.003). There was no relationship between sex and the degree of tooth retention (*p* = 0.206), the tooth position in the bone (*p* = 1.000), and the duration of the procedure (*p* = 0.650).

### 3.2. Influence of A-PRF Application on Postoperative Complications

#### 3.2.1. Pain and Analgesics Intake

**Figure 3:**
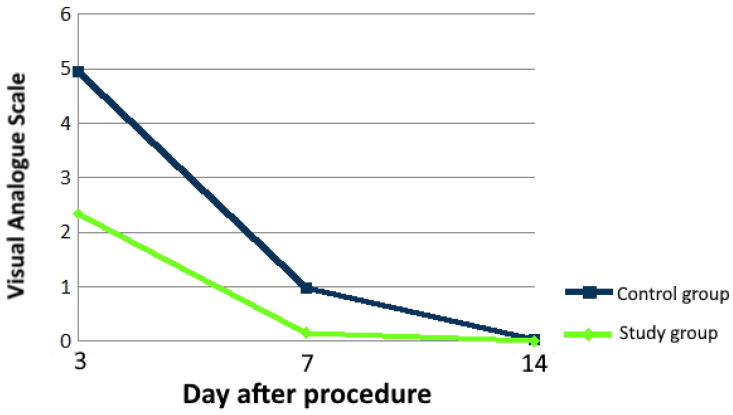
Time-dependent variability of pain intensity in the control and study groups.

The performed analysis of the research material showed significant association between the A-PRF application and pain intensity. It was observed that the pain was less intense in patients who had undergone the surgery with the inclusion of A-PRF use. The pain was significantly less intense in the study group on the 3rd postoperative day (*p* < 0.001) and on the 7th postoperative day (*p* < 0.001). On the 14th day, no significant differences were found (*p* > 0.05). On the 3rd day after the surgery, the median pain intensity, on a scale of 0 to 10, was 5 in the control group, whereas, in the study group, it was 2. On the 7th day after the surgery, the median pain intensity was 1 in the control group, whereas, in the study group, it was 0. There was no difference in pain intensity between the groups on the 14th day after the surgery (Figure 3).

**Figure 4:**
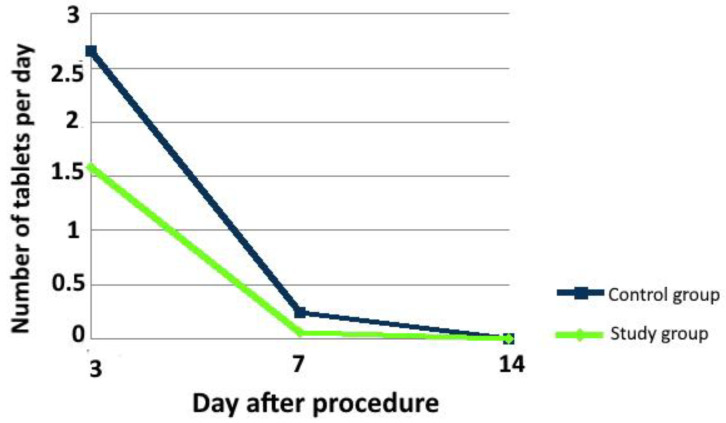
Time-dependent variability in the intake of painkillers in the control and study groups.

The intake of analgesics on the 3rd day after the surgery was strongly associated with A-PRF application. The analgesics intake was 3 tablets a day (1 tablet every 8 h) in the control group, whereas, in the study group, it was 2 tablets a day (1 tablet every 12 h; *p* < 0.001). On the 7th day after the procedure, patients in the control group took more drugs (*p* = 0.048). None of the patients were taking any painkillers on the 14th day after the surgery (*p* = 1.000; Figure 4).

A model of multivariate analysis of variance was performed, which examined the influence of A-PRF and selected variables (tooth position, degree of tooth retention, duration of the procedure) on postoperative pain (Supplementary Table S1). The application of A-PRF had a much more positive effect on the reduction in pain and lower consumption of NSAIDs than the more favorable position of the tooth on the 3rd and 7th day after the procedure. Tooth retention had no effect on the variability in the occurrence of intake. Additionally, the application of A-PRF had a more positive effect on the reduction in pain and lower consumption of NSAIDs than the shorter time of surgical procedure on the 3rd and 7th day after the procedure.

#### 3.2.2. Trismus

The research revealed that there is a statistical relationship between the A-PRF application and trismus severity on the 3rd (*p* < 0.001) and 7th day (*p* < 0.001) after the surgery. The severity of trismus in the study group was significantly lower than in the control group. There were no statistically significant differences between the groups on the 14th day after the surgery (*p* = 0.495; Table 1).

A model of multivariate analysis of variance was performed, which examined the influence of A-PRF and selected variables (tooth position, degree of tooth retention, duration of the procedure) on postoperative trismus (Supplementary Table S1). The application of A-PRF had a much more positive effect on the reduction in trismus than the more favorable position of the tooth on the 3rd and 7th day after the procedure. Tooth retention had no effect on the variability in the occurrence of trismus. Additionally, the application of A-PRF had a much more positive effect on the reduction in trismus than the shorter time of the surgical procedure on the 3rd and 7th day after the treatment.

#### 3.2.3. Edema

In case of the size of postoperative edema, there were statistically significant differences between the control group and the study group on the 3rd (*p* < 0.001) and 7th day (*p* < 0.001). Large edema was less frequent in the study group. There was no association between the use of A-PRF and the severity of edema on the 14th day after the procedure (*p* = 0.242; Table 1).

On the 3rd day in the control group, hard edema was more frequently noted (*p* = 0.006). On the 7th day in the control group, soft palpation affected 18 patients, and hard edema affected 8 patients. In the study group, 5 patients suffered from a soft edema, but no hard edema was found in any of the patients in this group (*p* < 0.001). On the 14th day after the surgery, no statistically significant differences were found between the groups (*p* = 0.242).

A model of multivariate analysis of variance was performed, which examined the influence of A-PRF and selected variables (tooth position, degree of tooth retention, duration of the procedure) on postoperative edema (Supplementary Table S1). The application of A-PRF had a much more positive effect on the reduction in edema texture than the more favorable position of the tooth on the 7th day after the procedure. The application of A-PRF had a much more positive effect on the reduction in edema size than the more favorable position of the tooth on the 3rd day after the procedure; the reverse was true on the 7th day. Similar results were obtained in the evaluation of the treatment time and the application of A-PRF. Tooth retention had no effect on the variability in the occurrence of edema.

#### 3.2.4. Followings Connected with Bleeding

The mean period of the time until the bleeding stopped (bleeding time—BT) after the procedure was analyzed. In the control group, it was 100 min (SD = 90 min). In the study group, the bleeding time was 68 min (SD = 60 min). The A-PRF socket filling was correlated with the bleeding time (*p* < 0.001). In the case of secondary bleeding, there was no association between A-PRF application and the bleeding after the achievement of primary hemostasis (*p* = 1.000).

Hematomas were statistically more frequently observed on 3rd day in patients without A-PRF socket filling (*p* < 0.001). The study shows that A-PRF protected against the onset of a hematoma. In the study group, the hematomas were not observed in the study group (Table 1).

A model of multivariate analysis of variance was performed, which examined the influence of A-PRF and selected variables (tooth position, degree of tooth retention, duration of the procedure) on postoperative hematoma and bleeding (Supplementary Table S1). The application of A-PRF had a much more positive effect on the reduction in hematomas than the more favorable position of the tooth on the 3rd and 7th day after the procedure. A-PRF reduced secondary bleeding on the 3rd day. Tooth retention had no effect on the variability in the occurrence of hematoma and bleeding.

#### 3.2.5. Prevalence of Dry Socket

In the analyzed material, no association between A-PRF use and dry socket prevalence was observed. On the 3rd day after the surgery, dry socket was observed in only 1 patient from the control group, whereas, in the study group, none of the patients suffered from dry socket (*p* = 1.000). On the 7th day after the surgery, dry socket was observed in 1 patient from the control group and in 1 patient from the study group (*p* = 1.000). On the 14th day after the surgery, none of the patients participating in the study complained of symptoms of dry socket (*p* = 1.000; Table 1).

A model of multivariate analysis of variance was performed, which examined the influence of A-PRF and selected variables (tooth position, degree of tooth retention, duration of the procedure) on postoperative alveolar osteitis (Supplementary Table S1). The application of A-PRF and tooth position had no effect on the reduction in dry socket. In contrast, A-PRF application and/or shorter time of surgery procedures reduce risk of alveolar osteitis on the 3rd, 7th, and 14th day. Tooth retention had no effect on the variability in the occurrence of hematoma and bleeding.

#### 3.2.6. Prevalence of Pyrexia

No association between A-PRF application and pyrexia in the post-operative period was observed. On the 3rd day after the surgery, 8 patients from the control group and 5 patients from the study group (*p* = 0.552) had elevated body temperature, whereas, on the 7th day after the surgery, only 1 patient from the control group had pyrexia (*p* = 1.000). On the 14th day after the surgery, none of the patients had pyrexia (*p* = 1.000; Table 1).

A model of multivariate analysis of variance was performed, which examined the influence of A-PRF and selected variables (tooth position, degree of tooth retention, duration of the procedure) on postoperative pyrexia (Supplementary Table S1). The application of A-PRF had a much more positive effect on the reduction in pyrexia than the more favorable position of the tooth on the 3rd day after the procedure. Additionally, the application of A-PRF had a much more positive effect on the reduction in pyrexia than the shorter time of surgery procedure on the 3rd day after the operation. In contrast, the time of surgery procedure was of greater importance on the 7th day. Tooth retention had no effect on the variability in the occurrence of hematoma and bleeding.

#### 3.2.7. Skin Warmth

During the follow-up examinations the prevalence of skin warmth in the vicinity of the post-operative area was assessed. The chi-square statistical test revealed the relation between A-PRF application and the prevalence of skin warmth. On the 3rd day after the surgery, skin warmth was observed in 13 patients from the control group and in 3 patients from the study group (*p* = 0.0141). On the 7th and 14th day after the surgery, no skin warmth was observed in either group (Table 1).

A model of multivariate analysis of variance was performed. It examined the influence of A-PRF and selected variables (tooth position, degree of tooth retention, duration of the procedure) on postoperative skin warmth (Supplementary Table S1). The application of A-PRF had a much more positive effect on the reduction in skin temperature than the more favorable position of the tooth on the 3rd day after the procedure. The application of A-PRF had a much more positive effect on the reduction in skin temperature than the shorter time of surgery procedure on the 3rd day after the procedure. On the 7th day, A-PRF and the time of surgery procedure had a much more positive effect on the reduction in skin warmth. Tooth retention had no effect on the variability in the occurrence of hematoma and bleeding.

## 4. Discussion

In the presented study, the analysis showed a significant relationship between the use of A-PRF and pain reduction on the 3rd and 7th postoperative day in the study group. It can be assumed that the pain was less intense. This is also confirmed by the lower dose of oral painkillers in the study. Kapse et al. [34] assessed postoperative pain on the VAS scale after the surgery in the group with and without autologous PRF. It was shown that the intensity of pain was lower in the study group on the 3rd and 7th postoperative day, and on the 14th day there was no evidence of the effect of PRF on the pain intensity [22]. In the study by Kumar et al. [35], which presented the pain on the VAS scale 1 day after third molar extraction, it was shown that the severity of pain was lower in the group of patients with sockets supplied with autologous PRF after surgery. The study by Dar et al. [36] showed a lower severity of pain in the group of patients in whom the alveolus was supplemented with PRF after tooth extraction, except the 14th day, when there was no significant difference. In a study by Trybek et al. [4], assessing pain using the NRS scale (numerical rating scale), it was shown that after the surgery, the severity of pain was lower in the group of patients with PRF sockets filling. Ozgul et al. [37] performed simultaneous extraction of both completely impacted third molars in the mandible, with similar difficulty of the procedure, by supplementing one alveolus, using PRF membrane. Patients were not informed on which side the fibrin clot had been placed. The patients assessed their pain intensity using the VAS scale. No effect of PRF on pain intensity was observed. The authors emphasize that the incorrect assessment of pain may have been influenced by the fact that the procedures in all patients were performed bilaterally during one visit [37]. Zahid and Nadershah [38] performed a similar study. A statistically significant reduction in the pain was observed in the A-PRF side compared with the controls. Gupta and Agarwal [39] described that pain on the 3rd postoperative day revealed considerable improvement on A-PRF sites as compared with control sites. In a study by Yüce and Kömerik [28], A-PRF application demonstrated rapid and continuous pain intensity reduction at each respective time point in comparison with the control. Caymaz and Uyanik [40] compared the postoperative effects of PRF, L-PRF (leukocyte- and platelet-rich fibrin), and A-PRF in terms of pain, swelling, and trismus after mandibular third molar surgery. The use of A-PRF after mandibular third molar extraction significantly reduces postoperative pain, and the patients of the A-PRF group needed to take less analgesics compared with the L-PRF group [30]. In the studies by Asutay et al. [41], Gülşen et al. [42], and Torul et al. [43], they did not confirm the statistically significant influence of PRF application on the pain intensity after the surgical extraction of wisdom teeth.

In this study, it was shown that the severity of edema on the 3rd and 7th postoperative day is significantly related to the use of A-PRF socket filling. Kapse et al. [34] assessed the edema used in the measurements of three distances between the outer corner of the eye and the angle of the mandible, from point T to point Ch and from point T to point Pg. The arithmetic sum of these distances, measured before the treatment, was subtracted from the sum of the distances measured at the control visit. In the observations made at the follow-up visits, the swelling was smaller when PRF was used to as a tool for the alveolus supplementation [34]. Kumar et al. [35] showed that the swelling in the 1st day after the surgery was characterized by lower severity in the group of patients with immediate PRF sockets supplementation just after the surgery. Dar et al. [36] measured the swelling between points T and Pg. The studies showed a lower severity of edema at each of the follow-up visits in the group of patients whose alveolus was supplemented with PRF after tooth extraction, except the 14th day, when there was no significant difference [36]. Trybek et al. [4] showed no relationship between edema and PRF application to the socket. Zahid and Nadershah [38] performed simultaneous extraction of both completely impacted third molars in the mandible, with similar difficulty of the procedure, by supplementing one of the alveoli using an A-PRF membrane. A statistically significant reduction in swelling was observed in the A-PRF side compared with the controls [38]. Similar results were obtained in the conducted analysis. In the study by Ozgul et al. [37], smaller horizontal edema was found in the study group on the 1st and 3rd day after the surgery, compared with the control group. Asutay et al. [41] and Gülşen et al. [42] did not show the effect of PRF on edema. Gupta and Agarwal [39] described that swelling on the 3rd postoperative day revealed considerable improvement on A-PRF sites as compared with the control sites. Torul et al. [43] proved that on the 7th postoperative day, edema was smaller in the study group with A-PRF application than in the control group.

Based on the analysis of the research material, it was found that A-PRF also has a significant impact on the occurrence of trismus and its intensity on the 3rd and 7th postoperative day. In the study group, on the 3rd and 7th day, the absence of trismus or the first degree of its severity was more often reported than in the control group. On the 14th day after the procedure, no statistically significant influence of A-PRF on the severity of post-treatment trismus was demonstrated. In the studies conducted by Kumar et al. [35], trismus showed a lower intensity in the group of patients in whom the alveoli were supplied with PRF in the 1st day after the surgery. Trybek et al. [4] also showed better mouth opening the in group with PRF. In the study by Asutay et al. [41] the effect of PRF application on the trismus was not confirmed. Gupta and Agarwal [39] described that trismus on the 3rd postoperative day revealed considerable improvement on A-PRF sites, as compared with the control sites.

In the analyzed material, no correlation was found between the A-PRF alveolus restoration and the incidence of dry socket. In the study by Eshghpour et al. [44], the frequency of dry socket was statistically lower in the group of patients in whom the extraction socket was supplemented with PRF. Unsal et al. [45] found dry socket in 8% of patients from the study group and in 18% of patients from the control group. They found no relationship between the use of PRF and the occurrence of symptoms of dry socket. Moreover, a significant correlation was demonstrated between the use of PRF and the occurrence of symptoms of dry socket in a group of smoking patients [45]. Kapse et al. [34] found no symptoms of dry socket in the group of patients with and without PRF. The same results were found in the studies by Asutay et al. [41].

In this study, we did not find any influence of A-PRF on the incidence of pyrexia in the postoperative period. After the surgery, during the follow-up visits, the incidence of excessive cheek skin warmth on the operated side was also assessed. Statistical analysis showed the relationship between the use of A-PRF and the incidence of skin warmth. Moreover, a statistically significant correlation was demonstrated between the use of A-PRF and the occurrence of hematoma on the 3rd day after the surgery. On the 7th and 14th day after the surgery, no correlation was observed between the use of A-PRF and the occurrence of hematoma within the surrounding tissues.

Various types of platelet-rich preparations have been used to stimulate wound healing after mandibular third molar extraction [31,32,33]. In our study, we used advanced platelet-rich fibrin which is characterized with gradual release and higher concentrations of growth factors than other rich platelet preparations [46]. In this study, only the cases where the mucoperiosteal flap with osteotomy was needed, and where the difficulty of surgical tooth extraction was assessed as moderately or very difficult, according to Pedersen’s classification, were included. As a result, cases were patients had moderate and difficult extraction of the third molar were included in the study. It helped us to unify the research targets. The study evaluates 13 clinical features before and after the surgical procedure. We analyzed the effect of platelet-rich fibrin on the occurrence of complications in the two groups, with and without A-PRF. The influence of other factors, such as the position of the tooth or the time of the surgical procedure, were excluded. The limitations of the study were heterogenicity in the number of women and men (prevalence of women) and the age. However, the median age in both groups was practically the same.

## 5. Conclusions

In our study we observed that the growth factors in A-PRF reduce postoperative complications, such as pain, trismus, edema, analgesics intake, presence of hematomas, and skin warmth, after mandibular wisdom teeth odontectomy. The study showed that A-PRF was more important than the position of the tooth or the duration of the procedure in reducing the incidence of postoperative complications. A-PRF is a valuable, safe autogenous material, and filling a socket with A-PRF may be considered as an efficient method of minimizing post-operative complications. This method can be easily used in clinical practice.

## Figures and Tables

**Table 1:** Characteristic of patients and clinical features (SD—standard deviation; *p*—*p* value).

Clinical and Pathological Patient Characteristics			Control Group	Study Group	*p*
Age Median ± SD (Range) [Years]			28.5 ± 5.7 (18–42)	29.3 ± 7.4 (19–47)	-
Gender		Female	32 (64%)	33 (66%)	0.216
		Male	18 (36%)	17 (34%)	
Grade of tooth retention		Complete retention	9 (18%)	3 (6%)	0.003
		Partial retention	41 (82%)	47 (94%)	
Tooth position		Vertical	25 (50%)	33 (66%)	0.839
		Horizontal	25 (50%)	17 (34%)	
Time of surgery procedure		<30 min	41 (82%)	40 (80%)	0.114
		≥30 min	9 (19%)	10 (20%)	
Pain (VAS scale)	3rd day	0–3	11 (22%)	42 (84%)	<0.001
		4–6	25 (50%)	8 (16%)	
		7–10	14 (28%)	0 (0%)	
	7th day	0–3	48 (96%)	49 (98%)	<0.001
		4–6	2 (4%)	1 (2%)	
		7–10	0 (0%)	0 (0%)	
	14th day	0–3	50 (100%)	50 (100%)	>0.050
		4–6	0 (0%)	0 (0%)	
		7–10	0 (0%)	0 (0%)	
Painkillers intake	3rd day	median (range)	3 (0–4) doses	2 (0–4)	<0.001
	7th day	median (range)	0 (0–2)	0 (0–2)	0.048
	14th day	median (range)	0 (0)	0 (0)	1.000
Trismus	3rd day	Lack of trismus	5 (10%)	23 (46%)	<0.001
		First grade	13 (26%)	22 (44%)	
		Second grade	21 (42%)	3 (6%)	
		Third grade	11 (22%)	1 (2%)	
	7th day	Lack of trismus	29 (58%)	45 (90%)	<0.001
		First grade	18 (36%)	5 (10%)	
		Second grade	3 (6%)	0 (%)	
		Third grade	0 (%)	0 (%)	
	14th day	Lack of trismus	50 (100%	50 (100%	0.495
		First grade	0 (%)	0 (%)	
		Second grade	0 (%)	0 (%)	
		Third grade	0 (%)	0 (%)	
Edema	Texture of edema				
	3rd day	Lack of edema	4 (8%)	12 (24%)	0.006
		Soft edema	33 (66%)	35 (70%)	
		Hard edema	13 (26%)	13 (26%)	
	7th day	Lack of edema	24 (48%)	45 (90%)	<0.001
		Soft edema	18 (36%)	5 (10%)	
		Hard edema	8 (16%)	0 (0%)	
	14th day	Lack of edema	47 (94%)	50 (100%)	0.242
		Soft edema	1 (2%)	0 (0%)	
		Hard edema	2 (4%)	0 (0%)	
	Size of edema				
	3rd day	Lack of edema	4 (8%)	12 (24%)	<0.001
		Small edema	17 (34%)	35 (70%)	
		Medium edema	27 (54%)	3 (6%)	
		Large edema	2 (4%)	0 (0%)	
	7th day	Lack of edema	24 (48%)	45 (90%)	<0.001
		Small edema	25 (50%)	5 (10%)	
		Medium edema	1 (2%)	0 (0%)	
		Large edema	0 (0%)	0 (0%)	
	14th day	Lack of edema	47 (94%)	50 (100%)	0.242
		Small edema	3 (6%)	0 (0%)	
		Medium edema	0 (0%)	0 (0%)	
		Large edema	0 (0%)	0 (0%)	
Bleeding time		<30 min	2 (4%)	21 (42%)	<0.001
		Up to 1 h	36 (72%)	22 (44%)	
		2–3 h	6 (12%)	5 (10%)	
		4–6 h	6 (12%)	2 (4%)	
Hematoma	3rd day	Lack of hematoma	35 (70%)	50 (100%)	<0.001
		Buccal hematoma	9 (18%)	0 (0%)	
		Submandible hematoma	5 (10%)	0 (0%)	
		Neck hematoma	1 (2%)	0 (0%)	
	7th day	Lack of hematoma	42 (84%)	47 (94%)	0.453
		Buccal hematoma	3 (6%)	1 (2%)	
		Submandible hematoma	3 (6%)	1 (2%)	
		Neck hematoma	2 (4%)	1 (2%)	
	14th day	Lack of hematoma	50 (100%)	50 (100%)	1.000
		Buccal hematoma	0 (0%)	0 (0%)	
		Submandible hematoma	0 (0%)	0 (0%)	
		Neck hematoma	0 (0%)	0 (0%)	
Secondary bleeding	3rd day	No	48 (96%)	50 (100%)	0.495
		Yes	2 (4%)	0 (0%)	
	7th day	No	50 (100%)	50 (100%)	1.000
		Yes	0 (0%)	0 (0%)	
	14th day	No	50 (100%)	50 (100%)	1.000
		Yes	0 (0%)	0 (0%)	
Dry socket	3rd day	No	49 (98%)	50 (100%)	1.000
		Yes	1 (2%)	0 (0%)	
	7th day	No	49 (98%)	49 (98%)	1.000
		Yes	1 (2%)	1 (2%)	
	14th day	No	50 (100%)	50 (100%)	1.000
		Yes	0 (0%)	0 (0%)	
Pyrexia	3rd day	No	42 (84%)	45 (90%)	0.552
		Yes	8 (16%)	5 (10%)	
	7th day	No	49 (98%)	50 (100%)	1.000
		Yes	1 (2%)	0 (0%)	
	14th day	No	50 (100%)	50 (100%)	1.000
		Yes	0 (0%)	0 (0%)	
Skin warmth	3rd day	No	37 (74%)	47 (94%)	0.014
		Yes	13 (26%)	3 (6%)	
	7th day	No	50 (100%)	50 (100%)	1.000
		Yes	0 (0%)	0 (0%)	
	14th day	No	50 (100%)	50 (100%)	1.000
		Yes	0 (0%)	0 (0%)	

## Data Availability

The data presented in this study are available on request from the corresponding author. The data are not publicly available due to privacy restrictions.

## Supplementary Materials

The following are available online at https://www.mdpi.com/article/10.3390/ijerph182413343/s1, Table S1: Influence of A-PRF application on postoperative complications depending on the tooth position, grade of retention and time of procedure (SS—sum-of-squares; DF—degrees of freedom; MS—mean squares; F—F ratio; *p*—*p* value).

## Abbreviations

A-PRF	advanced platelet-rich fibrin
Al	Alare
BMP-2	bone morphogenic factor 2
BMP-7	bone morphogenic factor 7
Ch	chelion
DF	degrees of freedom
F	F ratio
FGF	fibroblast growth factor
IL-1β	interleukin 1β
IL-4	interleukin 4
IL-6	interleukin 6
L-PRF	leukocyte and platelet-rich fibrin
MS	mean squares
*p*	*p* value
PD-EGF	platelet-derived epidermal growth factor
PDGF	platelet-derived growth factor
Pg	pogonion
PRP	platelet-rich plasma
SS	sum of squares
T	tragus
T0	time ‘zero’
TGF	transforming growth factor
TGFβ-1	tumor necrosis factor β-1
TNFα	tumor necrosis factor α
TSP-1	thrombospondin-1
VAS	visual analog scale
VEGF	vascular-endothelial growth factor
