# Supplementary material for: The Growth Factors in Advanced Platelet-Rich Fibrin (A-PRF) Reduce Postoperative Complications after Mandibular Third Molar Odontectomy
Source: Int J Environ Res Public Health. 2021 Dec 18;18(24):13343. doi: 10.3390/ijerph182413343 (PMC8702191; doi:10.3390/ijerph182413343)
Supplement: Supplementary file 1 [file ijerph-18-13343-s001.zip › ijerph-1476835-supplementary.pdf]

Supplementary Table S1. Influence of A-PRF application on postoperative complications depending on the tooth position, grade of retention and time of procedure (SS—sum-of-squares; DF—degrees of freedom; MS—mean squares; F—F ratio; *p*—*p* value).

		Statistics	SS	DF	MS	F	*p*	Observed power
		Tooth position (horizontal vs. vertical)						
Pain (VAS scale)	3rd day	A-PRF	156.001	1	156.001	42.619	<0.001	0.999
		Tooth position	0.597	1	0.597	0.163	0.687	0.069
		A-PRF and tooth position	0.036	1	0.036	0.921	0.921	0.051
	7th day	A-PRF	34.931	1	34.931	45.417	<0.001	0.983
		Tooth position	13.117	1	5.801	17.054	0.007	0.776
		A-PRF and tooth position	0.014	1	0.014	0.0188	0.891	0.520
	14th day	A-PRF	0.038	1	0.038	0.946	0.333	0.161
		Tooth position	0.038	1	0.038	0.946	0.333	0.161
		A-PRF and tooth position	0.038	1	0.038	0.946	0.333	0.161
Painkillers intake	3rd day	A-PRF	1124.602	1	1124.602	83.913	<0.001	1.000
		Tooth position	2.921	1	2.921	0.292	0.642	0.075
		A-PRF and tooth position	0.391	1	0.391	0.029	0.864	0.053
	7th day	A-PRF	196.170	1	196.170	8.256	0.005	0.812
		Tooth position	87.187	1	87.187	3.669	0.058	0.475
		A-PRF and tooth position	87.187	1	87.187	3.669	0.058	0.475
	14th day	no volatility						
Trismus	3rd day	A-PRF	10.469	1	10.470	12.860	< 0.001	0.944
		Tooth position	0.245	1	0.245	0.301	0.580	0.084
		A-PRF and tooth position	0.781	1	0.781	0.959	0.330	0.162

		Statistics	SS	DF	MS	F	*p*	Observed power
	7th day	A-PRF	65.264	1	65.264	8.350	< 0.001	0.820
		Tooth position	19.107	1	19.107	2.440	0.120	0.340
		A-PRF and tooth position	35.134	1	35.134	4.490	0.040	0.560
	14th day	A-PRF	0.340	1	0.340	1.974	0.160	0.285
		Tooth position	0.340	1	0.340	1.974	0.160	0.285
		A-PRF and tooth position	0.340	1	0.340	1.974	0.160	0.285
Edema texture	3rd day	A-PRF	1.921	1	1.921	1.880	0.174	0.274
		Tooth position	2.050	1	2.050	2.006	0.160	0.289
		A-PRF and tooth position	0.719	1	0.719	0.704	0.404	0.132
	7th day	A-PRF	58.086	1	58.086	70.740	< 0.001	1.000
		Tooth position	1.855	1	1.855	2.259	0.140	0.320
		A-PRF and tooth position	0.945	1	0.945	1.150	0.290	0.190
	14th day	A-PRF	0.151	1	0.151	2.560	0.110	0.350
		Tooth position	0.000	1	0.000	0.000	1.000	0.050
		A-PRF and tooth position	0.000	1	0.000	0.000	1.000	0.050
Edema size	3rd day	A-PRF	74.244	1	74.244	200.712	< 0.001	1.000
		Tooth position	0.063	1	0.063	0.171	0.689	0.069
		A-PRF and tooth position	1.232	1	1.232	3.331	0.071	0.439
	7th day	A-PRF	2.170	1	2.170	1.777	0.190	0.260
		Tooth position	7.843	1	7.843	6.422	0.010	0.710
		A-PRF and tooth position	2.800	1	2.800	2.293	0.130	0.320
	14th day	A-PRF	0.766	1	0.766	2.919	0.090	0.390
		Tooth position	0.085	1	0.085	0.324	0.570	0.090

		Statistics	SS	DF	MS	F	p	Observed power
		A-PRF and tooth position	0.085	1	0.085	0.324	0.570	0.090
Hematoma	3rd day	A-PRF	0.605	1	0.605	174.115	<0.001	1.000
		Tooth position	0.605	1	0.605	2.150	0.146	0.306
		A-PRF and tooth position	0.605	1	0.605	2.150	0.146	0.306
	7th day	A-PRF	39.970	1	39.970	142.750	<0.001	1.000
		Tooth position	0.766	1	0.766	2.740	0.100	0.370
		A-PRF and tooth position	0.766	1	0.766	2.740	0.100	0.370
	14th day	A-PRF	0.000	1	0.000			
		Tooth position	23.650	1	23.650			
		A-PRF and tooth position	0.000	1	0.000			
Secondary bleeding	3rd day	A-PRF	1850.244	1	1850.244	57.110	<0.001	1.000
		Tooth position	7.289	1	7.289	0.220	0.640	0.076
		A-PRF and tooth position	5.339	1	5.339	0.160	0.690	0.069
	7th day	no volatility						
	14th day	no volatility						
Dry socket	3rd day	A-PRF	0.168	1	0.168	2.958	0.088	0.398
		Tooth position	0.001	1	0.001	0.018	0.893	0.052
		A-PRF and tooth position	0.026	1	0.026	0.464	0.498	0.103
	7th day	A-PRF	0.009	1	0.009	0.946	0.333	0.161
		Tooth position	0.009	1	0.009	0.946	0.333	0.161
		A-PRF and tooth position	0.009	1	0.009	0.946	0.333	0.161
	14th day	no volatility						
Pyrexia	3rd day	A-PRF	69.003	1	69.003	86.140	< 0.001	1.000

		Statistics	SS	DF	MS	F	*p*	Observed power
		Tooth position	1.387	1	1.387	1.730	0.190	0.260
		A-PRF and tooth position	0.159	1	0.159	0.200	0.660	0.070
	7th day	A-PRF	0.002	1	0.002	0.105	0.745	0.061
		Tooth position	0.057	1	0.057	2.915	0.090	0.393
		A-PRF and tooth position	0.002	1	0.002	0.105	0.745	0.061
	14th day	no volatility						
Skin warmth	3rd day	A-PRF	12.951	1	12.951	143.903	< 0.001	1.000
		Tooth position	0.464	1	0.464	5.151	0.250	0.613
		A-PRF and tooth position	0.464	1	0.464	5.151	0.250	0.613
	7th day	A-PRF	314.120	1	314.120	770.368	< 0.001	1.000
		Tooth position	0.714	1	0.714	1.752	0.190	0.260
		A-PRF and tooth position	0.714	1	0.714	1.752	0.190	0.260
	14th day	no volatility						
Tooth retention was no volatility on post-operative complications								
Time of surgery procedure								
Pain (VAS scale)	3rd day	A-PRF	88.829	1	88.829	25.758	< 0.001	0.998
		Time of surgery procedure	18.591	1	18.591	5.391	0.022	0.632
		A-PRF and time of surgery procedure	1.877	1	1.877	0.544	0.462	0.113
	7th day	A-PRF	6.865	1	6.865	8.687	0.004	0.830
		Time of surgery procedure	2.675	1	2.675	3.385	0.068	0.445
		A-PRF and time of surgery procedure	1.018	1	1.018	1.288	0.259	0.202
	14th day	A-PRF	0.009	1	0.009	0.224	0.636	0.075

		Statistics	SS	DF	MS	F	p	Observed power
		Time of surgery procedure	0.009	1	0.009	0.224	0.636	0.075
		A-PRF and time of surgery procedure	0.009	1	0.009	0.224	0.636	0.075
Painkillers intake	3rd day	A-PRF	88.829	1	88.829	25.758	< 0.001	0.998
		Time of surgery procedure	18.591	1	18.591	5.391	0.022	0.632
		A-PRF and time of surgery procedure	1.877	1	1.877	0.544	0.462	0.113
	7th day	A-PRF	291.587	1	291.587	12.128	< 0.001	0.930
		Time of surgery procedure	81.860	1	81.860	3.405	0.070	0.450
		A-PRF and time of surgery procedure	81.860	1	81.860	3.405	0.070	0.450
	14th day	no volatility						
Trismus	3rd day	A-PRF	6.322	1	6.322	7.894	0.006	0.794
		Time of surgery procedure	2.191	1	2.191	2.736	0.101	0.373
		A-PRF and time of surgery procedure	0.034	1	0.034	0.042	0.836	0.054
	7th day	A-PRF	4.683	1	4.683	0.652	0.421	0.125
		Time of surgery procedure	23.060	1	23.060	3.214	0.076	0.426
		A-PRF and time of surgery procedure	86.577	1	86.577	12.067	< 0.001	0.930
	14th day	A-PRF	0.634	1	0.634	3.630	0.060	0.470
		Time of surgery procedure	0.259	1	0.259	1.490	0.230	0.230
		A-PRF and time of surgery procedure	0.259	1	0.259	1.490	0.230	0.230
**Edema**	**3rd day**	A-PRF	0.741	1	0.741	0.740	0.391	0.136

		Statistics	SS	DF	MS	F	p	Observed power
**texture**		Time of surgery procedure	3.583	1	3.583	3.578	0.061	0.465
		A-PRF and time of surgery procedure	0.898	1	0.898	0.897	0.345	0.155
	**7th day**	A-PRF	28.336	1	28.336	35.201	< 0.001	1.000
		Time of surgery procedure	2.222	1	2.222	2.760	0.100	0.380
		A-PRF and time of surgery procedure	2.097	1	2.097	2.605	0.110	0.360
	**14th day**	A-PRF	0.130	1	0.130	2.210	0.140	0,31
		Time of surgery procedure	0.005	1	0.005	0.090	0.760	0,06
		A-PRF and time of surgery procedure	0.005	1	0.005	0.090	0.760	0,06
**Edema size**	**3rd day**	A-PRF	38.877	1	38.877	106.322	< 0.001	1.000
		Time of surgery procedure	0.019	1	0.019	0.053	0.817	0.056
		A-PRF and time of surgery procedure	1.729	1	1.729	4.730	0.032	0.576
	**7th day**	A-PRF	0.183	1	0.183	0.139	0.710	0.070
		Time of surgery procedure	0.236	1	0.236	0.179	0.670	0.070
		A-PRF and time of surgery procedure	2.171	1	2.171	1.654	0.200	0.250
	**14th day**	A-PRF	0.883	1	0.883	3.380	0.070	0.440
		Time of surgery procedure	0.134	1	0.134	0.510	0.480	0.110
		A-PRF and time of surgery procedure	0.134	1	0.134	0.510	0.480	0.110
**Hematoma**	**3rd day**	A-PRF	31.917	1	31.917	108.193	< 0.001	1.000
		Time of surgery procedure	0.000	1	0.000	0.000	0.984	0.050

		Statistics	SS	DF	MS	F	p	Observed power
		A-PRF and time of surgery procedure	0.000	1	0.000	0.000	0.984	0.050
	**7th day**	A-PRF	28.249	1	28.249	95.925	< 0.001	1.000
		Time of surgery procedure	0.119	1	0.119	0.404	0.530	0.100
		A-PRF and time of surgery procedure	0.119	1	0.119	0.404	0.530	0.100
	**14th day**	A-PRF	15.355	1	15.355			
		Time of surgery procedure	0.000	1	0.000			
		A-PRF and time of surgery procedure	0.000	1	0.000			
**Dry socket**	**3rd day**	A-PRF	8.746	1	8.746	87.428	< 0.001	1.000
		Time of surgery procedure	0.008	1	0.008	0.081	0.775	0.059
		A-PRF and time of surgery procedure	0.008	1	0.008	0.081	0.775	0.059
	**7th day**	A-PRF	0.047	1	0.047	5.118	0.026	0.610
		Time of surgery procedure	0.047	1	0.047	5.118	0.026	0.610
		A-PRF and time of surgery procedure	0.047	1	0.047	5.118	0.026	0.610
	**14th day**	no volatility						
**Pyrexia**	**3rd day**	A-PRF	51.695	1	51.695	65.072	< 0.001	1.000
		Time of surgery procedure	1.808	1	1.808	2.276	0.134	0.320
		A-PRF and time of surgery procedure	0.266	1	0.266	0.335	0.563	0.088
	**7th day**	A-PRF	0.000	1	0.000	0.025	0.873	0.052
		Time of surgery procedure	0.171	1	0.171	9.181	0.003	0.850

		Statistics	SS	DF	MS	F	p	Observed power
		A-PRF and time of surgery procedure	0.000	1	0.000	0.025	0.873	0.052
	**14th day**	no volatility						
**Skin warmth**	**3rd day**	A-PRF	8.746	1	8.746	87.428	< 0.001	1.000
		Time of surgery procedure	0.008	1	0.008	0.081	0.775	0.059
		A-PRF and time of surgery procedure	0.008	1	0.008	0.081	0.775	0.059
	**7th day**	A-PRF	185.421	1	185.421	494.456	< 0.001	1.000
		Time of surgery procedure	2.159	1	2.159	5.758	0.020	0.660
		A-PRF and time of surgery procedure	2.159	1	2.159	5.758	0.020	0.660
	**14th day**	no volatility						
